# Recent trends in racial and regional disparities in cervical cancer incidence and mortality in United States

**DOI:** 10.1371/journal.pone.0172548

**Published:** 2017-02-24

**Authors:** Wonsuk Yoo, Sangmi Kim, Warner K. Huh, Sarah Dilley, Steven S. Coughlin, Edward E. Partridge, Yunmi Chung, Vivian Dicks, Jae-Kwan Lee, Sejong Bae

**Affiliations:** 1 Institute of Public and Preventive Health, Augusta University, Augusta, Georgia, United States of America; 2 Georgia Cancer Center, Augusta University, Augusta, Georgia, United States of America; 3 Department of Obstetrics and Gynecology, University of Alabama at Birmingham, Birmingham, Alabama, United States of America; 4 Department of Clinical and Digital Health Sciences, Augusta University, Augusta, Georgia, United States of America; 5 Department of Medicine, Emory University School of Medicine, Atlanta, Georgia, United States of America; 6 Department of Obstetrics and Gynecology, Korea University Guro Hospital, Seoul, Korea; 7 Division of Preventive Medicine and Comprehensive Cancer Center, University of Alabama at Birmingham, Birmingham, Alabama, United States of America; National Health Research Institutes, TAIWAN

## Abstract

**Background:**

Although black women experienced greater cervical cancer incidence and mortality rate reduction in recent years, they continue to have higher incidence rates than whites. Great variations also exist among geographic regions of the US, with the South having both the highest incidence and mortality rates compared to other regions. The present study explores the question of whether living in the South is associated with greater racial disparity in cervical cancer incidence and mortality by examining race- and region-specific rates and the trend between 2000 and 2012.

**Methods:**

The Surveillance, Epidemiology, and End Results (SEER) 18 Program data was used. Cervical cancer incidence and mortality rates, annual percent changes, and disparity ratios were calculated using SEER*Stat software and Joinpoint regression for four groups: US14-Non-Hispanic White (NHW), US14-Non-Hispanic Black (NHB), South-NHW, and South-NHB, where South included 4 registries from Georgia and Louisiana and US14 were 14 US registries except the four South registries.

**Results:**

The average age-adjusted cervical cancer incidence rate was the highest among South-NHBs (11.1) and mortality rate was the highest among US14-NHBs (5.4). In 2012, the degree of racial disparities between South-NHBs and South-NHWs was greater in terms of mortality rates (NHB:NHW = 1.80:1.35) than incidence rates (NHB:NHW = 1.45:1.15). While mortality disparity ratios decreased from 2000–2012 for US14-NHB (APC: -1.9(-2.3,-1.4), mortality disparity ratios for South-NHWs (although lower than NHBs) increased compared to US14-NHW. Incidence rates for NHBs continued to increase with increasing age, whereas rates for NHWs decreased after age 40. Mortality rates for NHBs dramatically increased at age 65 compared to a relatively stable trend for NHWs. The increasing racial disparity with increasing age in terms of cervical cancer incidence rates became more pronounced when corrected for hysterectomy prevalence.

**Conclusions:**

Black race and South region were associated with higher cervical cancer incidence and mortality. Cervical cancer rates uncorrected for hysterectomy may underestimate regional and racial disparities. Increasing incidence rates for older NHBs compared to NHWs warrant further research to determine whether screening should continue for NHBs over age 65.

## Introduction

It is estimated that in the United States (US), 12,990 women will be newly diagnosed with cervical cancer and 4,120 will die from the disease in 2016 [1]. Cervical cancer is the fourth most common female cancer worldwide with an estimated incidence of 528,000 cases and 266,000 deaths in 2012 [2]. In the US, cervical cancer incidence and death rates have dramatically declined since the introduction of the Papanicolaou (Pap) test, which detects precancerous changes and earlier stage cervical cancers[3]. As cervical cancer is the most prevalent Human Papillomavirus (HPV)-related malignancy, the introduction of the HPV vaccine in 2006 also shows promise of significant reduction in cervical cancer rates [4]. But it will need some time before reductions in cervical cancer are seen as a result. During the past decade (2002–2012) alone, cervical cancer incidence and mortality rates continued to decline annually by 2.4% and 0.9%, respectively. However, the progress has not been uniformly made across all racial/ethnic and regional groups [5–7].

Although black women experienced greater reduction in cervical cancer incidence rates at an average annual percent change (AAPC) of -3.0% over 2000–2009, compared to -1.9% for white women, black women continue to have higher cervical cancer incidence rates than whites (10.4 vs 7.8 per 100,000 persons) [5]. Black women also continued to have the highest cervical cancer mortality rate (4.3 per 100,000 persons) than any other racial/ethnic groups from 2000 to 2009 [5]. Great variations also exist among geographic regions of the US, with the South having both the highest incidence (8.5 per 100,000 persons for 2007–2011) and mortality rates (2.7 per 100,000 persons) compared to other regions [7]. The overall US cervical cancer incidence and mortality rates were 7.8 and 2.3 per 100,000, respectively [7]. While cervical cancer incidence rates continued to decline in the Northeast (APC = -2.7 [-4.8,-0.6]) and the West (APC = -2.8 [-4.7,-0.8]) regions between 2007 and 2011, no significant decrease was evident in the South (APC = -1.4 [-3.6,0.8]) during the same period [7].

Regional and racial/ethnic disparities in health outcomes are often associated with socioeconomic factors such as socioeconomic status [8], and neighborhood poverty [9]. According to the annual population report on income and poverty by the US Census Bureau, the South region continues to have the lowest median income and the highest poverty rate relative to the other regions [10]. Given that blacks disproportionately live in the South, these geographic variations raise a question about the combined effects of race and region on the outcomes of cervical cancer, specifically whether living in the South is associated with greater racial disparity in cervical cancer incidence and mortality. The present study explores the question by examining race- and region-specific cervical cancer incidence and mortality rates and changes between 2000 and 2012.

## Methods

### Data

Incidence and mortality data of cervical cancer were obtained from Surveillance, Epidemiology, and End Results (SEER) 18 Program, supported by the National Cancer Institute. SEER collects cancer incidence and survival data from population-based cancer registries throughout the US. Cervical cancer cases (specifically, site recode ICD-O-3/WHO 2008 = ‘Cervix Uteri,’ corresponding to C530-C539) were obtained from the SEER18 database, which comprises 18 registries representing approximately 28% of the US population (Connecticut; Hawaii; Iowa; New Mexico; Utah; California excluding San Francisco, San Jose-Monterey, and Los Angeles; Kentucky; Louisiana; New Jersey; San Francisco-Oakland Metropolitan Statistical Area, California; Metropolitan Detroit, Michigan; Seattle (Puget Sound), Washington; Metropolitan Atlanta, Georgia; San Jose-Monterey, California; Los Angeles, California; Alaska Natives; Rural Georgia; and Greater Georgia) based on the US 2010 census, and include the largest geographic coverage compared to SEER 9 and SEER 13 data [11].

For the purpose of this study, four (Louisiana; Metropolitan Atlanta, Georgia; Rural Georgia; and Greater Georgia) of the 18 registries of SEER 18 data, were categorized into the South region, and the remaining 14 registries were categorized as the US14 region, as described in previous health disparity literature [12]. Case counts and the population at risk were selected among women aged 20 years and older and stratified by age in 5-year intervals (20–24, 25–29, 30–34, 35–39, 40–44, 45–49, 50–54, 55–59, 60–64, 65–69, 70–74, 75–79, 80–84, 85 or older), year of diagnosis (2000–2012), region (South, US14), ethnicity (Hispanic and Non-Hispanic), and race (White and Black). Four groups were formulated based on combination of two race/ethnic groups of Non-Hispanic White (NHW) and Non-Hispanic Black (NHB) and two region/registry groups (South and US14): US14-NHW, US14-NHB, South-NHW, and South-NHB. All data were accessed using SEER*Stat, version 8.3.2 [13].

The Behavioral Risk Factor Surveillance System (BRFSS) data were used to generate survey weighted estimates of hysterectomy prevalence in the US through the National Center for Health Statistics Website. BRFSS is an ongoing, state-based, random-digit-dialing annual telephone survey of health behavior among noninstitutionalized US civilian population aged 18 years or older. Annual BRFSS data has questions on cervical cancer screening including hysterectomy status (“Have you had an operation to remove the uterus or womb”). In order for BRFSS data to directly correspond to SEER 18 data, hysterectomy status was collected by year of diagnosis for five-year span (2008 to 2012), thirteen BRFSS states directly corresponding to SEER registry data (Georgia, Louisiana, Alaska, California, Connecticut, Hawaii, Iowa, Kentucky, Michigan, New Jersey, New Mexico, Washington, and Utah), and the same 5-year age increments from 20 years to ≥85 years [14].

### Statistical analysis

Age-adjusted incidence and mortality rates of cervical cancer were calculated as cases per 100,000 women on the basis of the 2000 US Standard Population using SEER*Stat software, and corresponding 95% confidence intervals (CIs) were calculated based on a modified gamma distribution [15]. Incidence rates less than 15 cases and mortality rates less than 10 deaths for the time interval were suppressed. Annual percent changes (APCs) and their 95% CIs were calculated to characterize trends in cancer rates over time by fitting a least squares linear regression line on the natural logarithm of the annual age-adjusted rates (on a log-linear model) using the calendar year as the predictor variable to characterize temporal trends in cervical cancer incidence rates. In order to evaluate the increasing or decreasing trends for incidence and mortality rates as well as disparity ratios over a thirteen-year span, APCs and corresponding 95% CIs between 2000 and 2012 were reported for the four groups defined earlier: US14-NHW, US14-NHB, South-NHW, and South-NHB. Each APC was tested on the hypothesis that an APC does not change over time (i.e., APC = 0) using exact probabilities from t-distribution. Log-linear Joinpoint regression models were used to examine trends across year at diagnosis by estimating the number of joinpoints (like change-points) for a series of joined and straight lines to the trends in rates of cervical cancer [16]. Up to 3 joinpoints were allowed in the models.

For relative evaluation of disparities in cervical cancer among four groups, incidence and mortality disparity ratios were computed by dividing race- and region-specific cervical cancer incidence and mortality rates for each of US14-NHB, South-NHW, and South-NHB by the rates of US14-NHW (a reference group). Age-specific analysis was performed to further understand regional and racial differences in cervical cancer incidence and mortality rates. For this query, age segments included five categories of 25–29, 30–49, 50–64, 65–74, and 75 years or older. US14-NHW group was used as reference group to calculate disparity ratios for US14-NHB, South-NHW and South-NHB by year (2000–2012) and five-category age-specific groups. Finally, age-specific incidence and mortality rates of cervical cancer were examined for NHW and NHB women in the South.

The corrected incidence and mortality rates of cervical cancer were calculated by removing age-, race-, and regional-specific hysterectomy prevalence estimates (i.e., the proportion of women who no longer have a cervix) from the denominator of the census population. The hysterectomy corrected population is, *P*_*c*_ = *P* × (1 − *h*), where *P* is the census population, *P*_*c*_ is the population size corrected for hysterectomy, and *h* is the hysterectomy prevalence. The data procedure has been published in detail elsewhere, but is briefly summarized here [17]. The overall age-standardized rates of cervical cancer for women aged ≥20 years were calculated using the standard 2000 US census population for both the uncorrected denominator and hysterectomy corrected denominator. Race and region stratified average annual age-specific cervical cancer incidence rates (*I*_*c*_) and corresponding 95% CI were then calculated using the hysterectomy-corrected number at risk, $I_{c}=\left(\frac{n}{P_{c}}\right)\times 100,000$.

The SEER Joinpoint software version 4.3.1.0 (April 2016) was used for all trend analyses in this study [16]. All other statistical analyses except SEER*Stat and SEER Joinpoint software were conducted using Statistical Analysis Software version 9.4 (SAS Institute, Cary, NC).

## Results

Age-adjusted incidence and mortality rates of cervical cancer and APC over a thirteen-year span from 2000 to 2012 are presented in Table 1. Overall, the incidence rate decreased from 9.6 per 100,000 in 2000 to 7.4 per 100,000 in 2012 with an average annual decrease of 1.9% (95% CI = [-2.3, -1.5]). The rate of decrease was similar between US14 and the South. The South had a persistently higher incidence of cervical cancer than US14. Compared with NHW women, NHB women experienced a sharper decrease in incidence (APC = -2.9% vs. -1.0% per year) in both regions, but NHB remained as having a higher incidence in 2012 (9.0 per 100,000 versus 6.8 per 100,000). On average, the cervical cancer mortality rate in US decreased by 1.5% (95% CI = [-1.9, -1.1]) annually between 2000 and 2012. The overall trend of reduction was largely accounted for by the decrease in mortality rate experienced in US14 (APC = -1.5, 95% CI = [-2.0, -1.1]). There was no significant trend of reduction in the South (APC = -0.2, 95% CI = [-1.0, 0.6]) during this period. Although overall there was no significant decreasing trend in the South, the mortality rate among South-NHB significantly reduced from 4.4 per 100,000 in 2000 to 3.6 per 100,000 in 2012 (APC = -1.9, 95% CI = [-3.7, -0.1]), and to a lesser extent compared to US14-NHB (APC = -2.6, 95% CI = [-3.2, -2.0]).

**Table 1:** Trends in incidence and mortality rates by race and region.

Type		Race & Ethnicity	Year of diagnosis								APC
			2000	2002	2004	2006	2008	2010	2012	2000–2012	2000–2012
**Age-adjusted Rates^**1**^**	**Incidence**	**ALL**	9.6	8.9	8.3	8.2	8.2	7.6	7.4	8.2	-1.9*(-2.3, -1.5)
		**US14**	9.5	8.7	8.2	8.2	7.9	7.5	7.3	8.1	-1.9*(-2.3, -1.6)
		**South**	10.4	9.8	8.6	8.1	9.4	8.1	8.1	8.8	-1.8*(-2.6, -0.9)
		**NHW**	8.1	7.8	7.2	7.3	7.4	7.0	6.8	7.3	-1.0*(-1.6, -0.5)
		**NHB**	12.7	11.7	11.1	9.8	10.9	9.5	9.0	10.5	-2.9*(-3.6, -2.1)
		**US14-NHW**	7.9	7.6	7.2	7.2	7.1	6.9	6.6	7.1	-1.1* (-1.7, -0.5)
		**US14-NHB**	12.3	10.4	10.7	9.5	9.8	9.4	8.6	10.0	-2.9* (-3.7, -2.1)
		**South-NHW**	9.2	8.6	7.4	7.4	8.8	7.8	7.6	8.2	-0.9 (-1.9, 0.1)
		**South-NHB**	13.4	13.6	11.9	10.2	12.5	9.7	9.6	11.1	-2.9* (-4.1, -1.8)
	**Mortality^**2**^**	**ALL**	2.8	2.6	2.4	2.4	2.4	2.3	2.3	2.8	-1.5*(-1.9, -1.1)
		**US14**	2.8	2.5	2.4	2.4	2.3	2.2	2.3	2.7	-1.5*(-2.0,-1.1)
		**South**	2.8	3.0	2.8	2.9	3.0	2.7	2.9	3.1	-0.2 (-1.0, 0.6)
		**NHW**	2.3	2.2	2.1	2.1	2.1	2.0	2.1	2.4	-1.0*(-1.5, -0.6)
		**NHB**	4.4	5.0	5.6	4.9	5.0	3.7	3.6	5.4	-1.9*(-3.7, -0.1)
		**US14-NHW**	2.3	2.2	2.1	2.1	2.0	2.0	2.0	2.4	-1.1* (-1.6, -0.7)
		**US14-NHB**	5.6	5.1	4.5	4.4	4.2	4.0	3.8	5.4	-2.6* (-3.2, -2.0)
		**South-NHW**	2.3	2.4	2.0	2.3	2.5	2.6	2.7	2.4	1.1 (-0.1, 2.3)
		**South-NHB**	4.4	5.0	5.6	4.9	5.0	3.7	3.6	5.2	-1.9* (-3.7, -0.1)
**Disparity Ratios**	**Incidence**	**US14-NHW**	1	1	1	1	1	1	1	1	Reference group
		**US14-NHB**	1.56	1.37	1.49	1.32	1.38	1.36	1.30	1.41	-1.1*(-2.0, -0.3)
		**South-NHW**	1.16	1.13	1.03	1.03	1.24	1.13	1.15	1.15	0.3 (-1.0, 1.5)
		**South-NHB**	1.70	1.79	1.65	1.42	1.76	1.41	1.45	1.56	-1.6 (-3.1, 0.1)
	**Mortality**	**US14-NHW**	1	1	1	1	1	1	1	1	Reference group
		**US14-NHB**	2.43	2.32	2.14	2.09	2.10	2.00	1.90	2.25	-1.9*(-2.3, -1.4)
		**South-NHW**	1.00	1.09	0.95	1.10	1.25	1.30	1.35	1.00	2.8*(1.4, 4.1)
		**South-NHB**	1.81	2.27	2.67	2.33	2.50	1.85	1.80	2.17	-0.9 (-4.0, 2.4)

^1^ Rates are per 100,000 and age-adjusted to the 2000 US Standard Population (19 age groups–Census P25-1130) standard. Confidence intervals (Tiwari mod) are 95% for APC.

^2^ Underlying mortality data provided by National Center for Health Statistics (NCHS)—CDC.

*Statistically significant at 5% level.

Abbreviations: NHW–non-Hispanic white, NHB–non-Hispanic black, US14 –SEER18 registries excluding the 4 southern registries, APC–Annual Percent Change.

**Fig 1:**
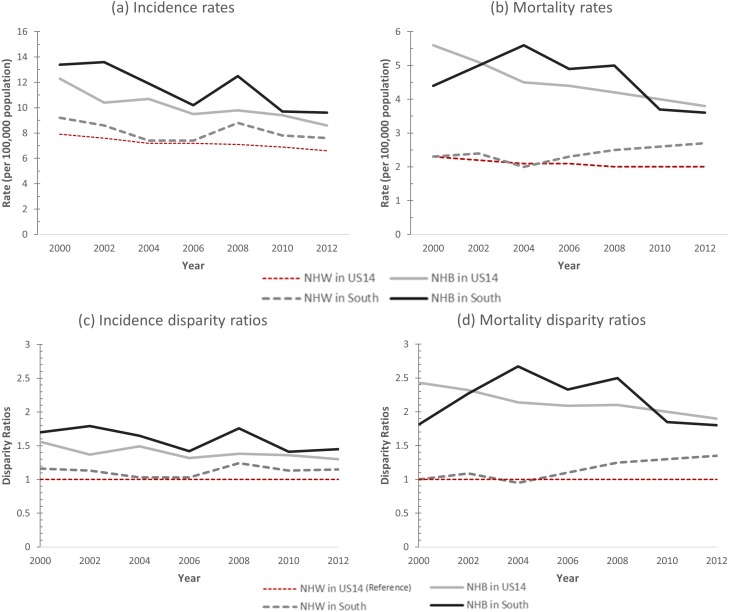
Cervical cancer incidence and mortality rates and disparity ratios by year, region, and race. (A) Age-adjusted cervical cancer incidence rate of US14-NHW, US14-NHB, South-NHW, and South-NHB women by year of diagnosis. (B) Age-adjusted cervical cancer mortality rates of cervical cancer of US14-NHW, US14-NHB, South-NHW, and South-NHB women by year of diagnosis. (C) Incidence disparity ratios of US14-NHB, South-NHW, and South-NHB women by year of diagnosis with US14-NHW as reference group. (D) Mortality disparity ratios of US14-NHB, South-NHW, and South-NHB women by year of diagnosis with US14-NHW as reference group. Abbreviations: NHW–non-Hispanic white, NHB–non-Hispanic black, US14 –SEER18 registries excluding the 4 southern registries.

Overall, US14-NHW had the lowest incidence and mortality rates throughout the period (Fig 1A and 1B). When disparity ratios were computed using the best performing group as the reference, disparity ratios in cervical cancer incidence progressively decreased between 2000 and 2012 for NHB women in both regions (APC = -1.1, 95% CI = [-2.0, -0.3] in US14, APC = -1.6, 95% CI = [-3.1, 0.1] in South), but disparity ratios for South-NHW remained stable (APC = 0.3, 95% CI = [-1.0, 1.5]) as seen in Fig 1C. The mortality disparity ratio followed a similar, but more pronounced, trend (Fig 1D). While disparity in mortality narrowed for NHB women, particularly for US14-NHB (mortality disparity ratio = 2.4 in 2000 vs. 1.9 in 2012, APC = -1.9 and 95% CI = [-2.3, -1.4]), a previously nonexistent disparity emerged for South-NHW (mortality disparity ratio = 1.0 in 2000 vs. 1.4 in 2012, APC = 2.8 and 95% CI = [1.4, 4.1]) as compared to US14-NHW. Overall, when US14-NHW was set as a reference group, the incidence and mortality ratios of black women in both regions were much higher. Disparities in mortality rates for South-NHW increased between 2000 and 2012 even though incidence disparities remained stable during same time span (Table 1).

**Fig 2:**
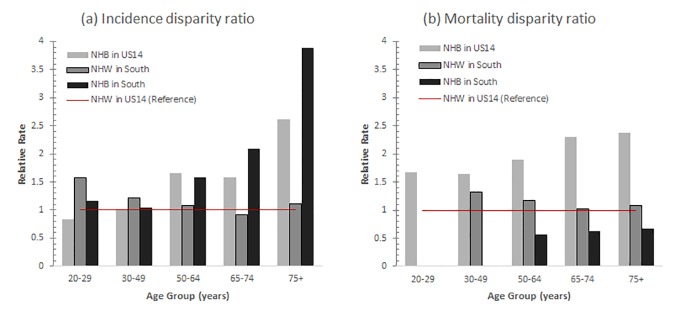
**Trends of age-specific cervical cancer (A) incidence and (B) mortality disparity ratios by region between 2000 and 2012 for US14-NHW, US14-NHB, South-NHW, and South-NHB.** Abbreviations: NHW–non-Hispanic white, NHB–non-Hispanic black, US14 –SEER18 registries excluding the 4 southern registries.

Age-specific analysis was performed to further understand regional and racial disparities in cervical cancer incidence and mortality rates. Fig 2 shows age-adjusted rates and disparity ratios of cervical cancer stratified by age-specific groups of 20–29, 30–49, 50–64, 65–74, and 75 years and older for the four race/region groups (US14-NHW, US14-NHB, South-NHW, and South-NHB). Regardless of region, age-adjusted incidence rates increased with increase in age for NHB but decreased for NHW after age 50, while age-adjusted mortality rates increased with increase in age for all four groups. As seen in Fig 2, incidence disparity ratios between NHW and NHB are relatively similar until age of 49 years, but after the age of 50 years racial difference in incidence disparity ratio can be observed. The incidence rates for South-NHB compared to US14-NHW (reference) are higher by 1.5 times (1 vs 1.59) among age group 50–64 years, around 2 times higher (1 vs 2.09) among age group 65–74 years, and more than three times higher (1 vs 3.88) among age group 75 years and older. Furthermore, there is a regional disparity in incidence rates for NHB women; for all age groups, South-NHB consistently had higher incidence rates than US14-NHB. The mortality disparity ratio for all age groups was the highest for US14-NHB, followed by South-NHW, US14-NHW, and South-NHB.

**Fig 3:**
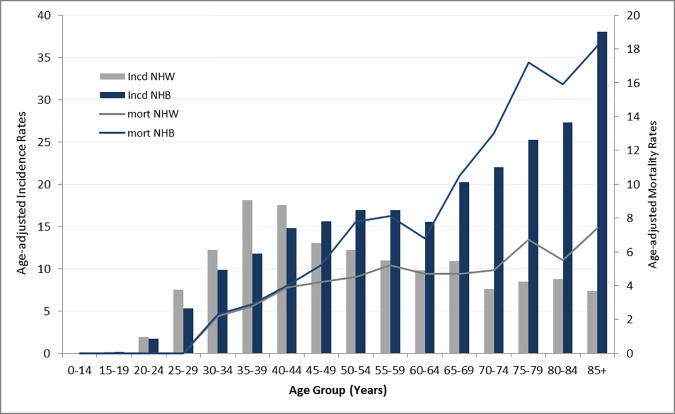
Age-specific cervical cancer incidence and mortality rates in the South by race (2008–2012). Bar graphs represent incidences rates (gray for NHW and navy for NHB) and line graphs represent mortality rates (gray for NHW and navy for NHB).Abbreviations: NHW–non-Hispanic white, NHB–non-Hispanic black.

Fig 3 shows age-specific incidence and mortality rates of cervical cancer in the South for the most recent five-year period (2008–2012) to further understand regional and racial differences in cervical cancer incidence and mortality rates. When we compared age-specific distributions of cervical cancer incidence and mortality rates between South-NHB and South-NHW, South-NHB women had lower incidence rates in younger ages (by age 44 years). The incidence rates among South-NHW increased until age 44 years and then decreased, while for South-NHB there was a steady increasing trend with increasing age. The incidence rates of age group 65–69 years for South-NHB were twice that of South-NHW (20.2 vs. 10.9), and the difference became even greater with increasing age. For age group 85 years and over, the incidence rates for NHB were almost five times that of NHW women (South-NHW: 7.4 and South-NHB: 38.0). Similarly, the mortality rates were similar between NHW and NHB women in younger age groups (<44 years), and the racial disparities became greater with increasing age. The mortality rates for NHB women showed a sharper increase with increasing age compared to NHW women, reaching a 2.5-fold difference among those 85 years and older (South-NHW: 7.4 and South-NHB:18.2). Age-specific incidence and mortality rates for US14 showed a similar pattern to those in the South. The incidence rates among NHW women increased by age 44 years and then decreased, while for NHB women there was a steady increasing trend. The mortality rates for NHB women also showed a sharp increase with advancing age, reaching a 3-fold difference (5.4 for US14-NHW and 16.3 for US14-NHB) among those 85 years and older (S1 Table).

**Fig 4:**
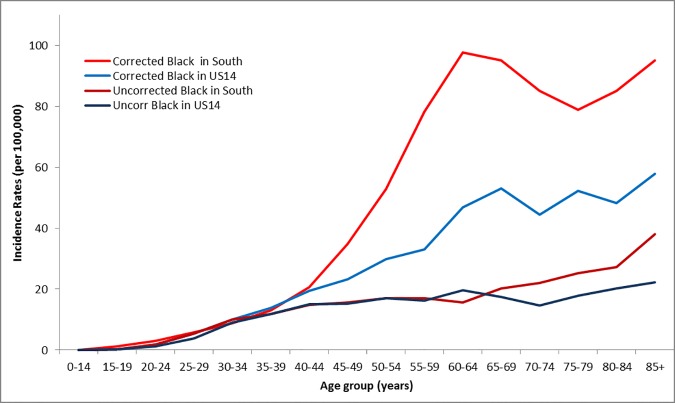
Age-specific cervical cancer incidence rates of black women pre- and post- US hysterectomy prevalence rate correction (2008–2012). Abbreviations: NHB–non-Hispanic black.

Age-specific incidence of cervical cancer for NHB women after correcting for hysterectomy prevalence in the US were examined further, as Rositch and colleagues reported [17]. The corrected incidence rates of cervical cancer were calculated by removing women who have had hysterectomy from the denominator population. The hysterectomy information came from Behavioral Risk Factor Surveillance System (2008 through 2012). Fig 4 illustrates age-specific cervical cancer incidence rate trends for NHB women based on hysterectomy-correction status (corrected and uncorrected) and regions (South and US14). All four trends (uncorrected-South, corrected-South, uncorrected-US14, and corrected-US14) show similar rates until age group 35–39 years. Uncorrected rates for NHB women in the South and US14 show a slight increase until age 64 and remain similar, but after age 65 the incidence rates in the South are higher than that of US14 with the biggest margin observed at age ≥ 85 (38 in South vs 22.2 in US14). Once the cervical cancer incidence rates are corrected for hysterectomy, a different trend is observed. After age 40, the hysterectomy-corrected incidence rates in both regions show a sharp increase and reach the highest point at age 60–64. For South-NHB, corrected incidence rate decreases between age 65–74 and increases again from age 80, while for US14-NHB rates remain relatively stable. For age groups 40–44 and older, corrected incidences are higher among South-NHB than US14-NHB. As seen in Fig 4, regional disparity exists in South region and the disparity becomes even greater when considering hysterectomy prevalence in incidence rates.

## Discussion

We observed a decrease in racial disparity in recent years. Racial disparities in cervical cancer have been well recognized [5, 9]. Previous studies have suggested a complex interplay of biological, socioeconomic and cultural factors underlying the phenomenon. Compared with whites, black women had lower relative survival rates, and the difference was largely accounted for by presentation of disease at later stages, squamous histology and mode of treatment [18]. Some evidence exists that black women are less likely to receive optimal treatment due to patient refusal, lack of appropriate physician recommendation for treatment, and poorer health and comorbid conditions [19].

However, according to data from Saslow et al., there was no racial variation in the use of screening among women ages 21–65 years, the prime age group for cervical cancer screening under the current national guidelines [20]. Other studies have shown that prevalence of Pap test use was slightly higher among black women with 84.7% versus 83.1% in non-Hispanic whites [14], and adherence to follow-up after an abnormal Pap test was not different between racial groups [21]. However, as we and others [17] observed, more black women are diagnosed at older ages than white women, comparable use of the Pap test under the current age recommendation may have limited impact on reducing the racial disparity. In this regard, it is notable that black women aged 65 or older were less likely to receive Pap test than their white or Hispanic peers (48% versus 64% and 53%) in a national survey [22]. More research is needed to develop personalized screening recommendations based on individual risk factors.

Cervical cancer incidence in the United States has decreased significantly since the adoption of the Pap test and HPV testing, and there is a significant inverse correlation between rates of cervical cancer screening and cervical cancer incidence. However, research has not demonstrated lower rates of screening among black women ages 21–65 years [20]. In fact, some studies have shown that prevalence of Pap test use was slightly higher among black women, with 84.7% versus 83.1% in non-Hispanic whites [14], and adherence to follow-up after an abnormal Pap test was not different between racial groups [21]. However, as we and others [17] have observed, more black women are diagnosed at older ages than white women. While United States Preventive Task Force (USPSTF) and American Society for Colposcopy and Cervical Pathology (ASCCP) guidelines [20, 23] do not recommend routine cervical cancer screening after age 65 if they have had negative tests in the decade before screening stopped, more research is warranted to determine whether screening should continue for black women over age 65 and to determine whether they qualify for discontinuation of screening.

Investigation on age-specific cervical cancer incidence and mortality rates in the South by race revealed that racial disparities in incidence and mortality rates become even greater after 40. Incidence rates of white women decrease after age 40 while those of black women increase with increasing age. It is interesting that disparity is the greatest in both incidence and mortality at age ≥ 85. Based on our results, older black women living in the South might be a target population to reduce incidence and mortality of cervical cancer, and the high disparity among women aged >85 might be due to lack of screening and current screening guidelines. These incidence disparities were even larger when incidence rates were calculated by removing women that have had hysterectomy from the denominator population. Although every hysterectomy performed does not necessarily eliminate the possibility of developing cervical cancer, uncorrected incidence and mortality rates of cervical cancer might underestimate regional and racial disparities. Even though HPV prevalence has been reduced among young women following HPV vaccine introduction in the US [4], the recommendation of HPV vaccination for young girls and women will not reduce cervical cancer among older women for several years. Nevertheless, ensuring that black and other eligible girls and young women have access to HPV vaccination is likely to reduce the burden of cervical cancer and disparities in the disease in future years.

We observed that geographic setting, beyond race, was also associated with cervical cancer incidence and, to a greater extent, mortality from the disease. Specifically, white young women in the South were more likely to have cervical cancer and die from the disease than their counterparts in other US regions. In the South, women of all races are affected by the disproportionately high rates of poverty, one of the most pervasive and consistent indicators of poor health [24]. Geographic variations in health may reflect the presence, or lack thereof, of social and public services and health infrastructure and sociocultural resources supporting health-related experiences [25]. Health insurance coverage is among the important factors affecting cervical cancer screening [26–28]. Adequate therapeutic intervention after abnormal findings is not only related to individual factors such as race and poverty but also is directly impacted by macro-level policies [19, 29–31]. A recent analysis of nonelderly cancer patients registered in the SEER data indicates that residence in a Southern registry was a significant independent predictor of lack of health insurance. Medicaid rates were also highest in the Southern registries [32]. The passage of the Patient Protection and Affordable Care Act in 2010 created the opportunity for states to expand Medicaid to cover low income families and individuals who are otherwise uninsured or underinsured; however, 19 states, including 10 states in the South, have opted out of Medicaid expansion. Compared with non-expansion states, (which include Louisiana prior to July of 2016 and Georgia, as represented in our study), expansion states have demonstrated a greater decrease in the uninsured population and a significant increase in colorectal and cervical cancer screening particularly among low-income patients [33]. Considering nearly 90% of people in the coverage gap reside in the South[34], these early findings suggest a positive impact towards reducing health disparity can be made by adoption of Medicaid expansion in the Southern states.

This analysis is not without limitations. First, individual-level socioeconomic status information was not available in our study. Adjustment for socioeconomic status in the assessment of health disparity could have been informative to characterize the nature of racial and geographic disparity. Healthcare in geographically underserved areas is often complicated by distrust and/or perceived or real discrimination and may play a significant role in the delay in seeking treatment; thus raising the likelihood of increased morbidity and mortality. Furthermore, religious beliefs, or the lack of transportation could contribute to a delay in seeking care. Furthermore, counties and agencies may lack the adequate funding at the state level to provide necessary educational material or healthcare within the population, especially since Medicaid expansion was optional for the states surveyed [35]. Moreover, many women may not seek treatment out of fear, lack of emotional support or may not have a regular provider [36]. Second, our analysis spanned a relatively short time period of 13 years (2000–2012), and thus, it would be interesting to see the results of regional and racial disparities if we add more years to our analysis. Since there are only two Southern states in the SEER program (Georgia and Louisiana), one should be cautious when generalizing to the entire South. Lastly, although not a limitation *per se*, our focus on the interplay of black race and Southern residence led to the exclusion of minority groups other than black race in the analysis, who have received due attention in other disparity literature [28, 37]. In summary, the present analysis of SEER data from 2000–2012 suggests continuing progress during a recent decade in terms of racial disparity but a stalled improvement in cervical cancer incidence and mortality in terms of regional disparity, particularly among young white women in the South. Because of the small numbers in age-, race- and region-specific analysis, the latter finding should require future validation.

## Supporting information

S1 Table Age-specific incidence and mortality rates stratified by region (South versus US14) and race/ethnicity (Non-Hispanic White and Non-Hispanic Black). (DOCX)
