# Supplementary material for: Recent trends in racial and regional disparities in cervical cancer incidence and mortality in United States
Source: PLoS One. 2017 Feb 24;12(2):e0172548. doi: 10.1371/journal.pone.0172548 (PMC5325259; doi:10.1371/journal.pone.0172548)
Supplement: S1 Table — (DOCX) [file pone.0172548.s001.docx]

Supplementary Table 1. Age-specific incidence and mortality rates stratified by region (South versus US14) and race/ethnicity (Non-Hispanic White and Non-Hispanic Black).

| Age-specific groups | Incidence rates^1^ | | | | Mortality rates^2^ | | | |
| --- | --- | --- | --- | --- | --- | --- | --- | --- |
|  | NHW US14 | NHB  US14 | NHW South | NHB South | NHW US14 | NHB  US14 | NHW South | NHB South |
| <20 | 0.2 | 0.1 | 0.2 | 0.1 | 0.0 | 0.0 | 0.0 | 0.0 |
| 20-24 | 1.2 | 1.2 | 1.9 | 1.7 | 0.1 | 0.2 | 0.0 | 0.0 |
| 25-29 | 5.0 | 3.8 | 7.5 | 5.3 | 0.5 | 0.8 | 0.0 | 0.0 |
| 30-34 | 10.3 | 8.9 | 12.2 | 9.9 | 1.2 | 1.8 | 2.2 | 2.3 |
| 35-39 | 13.8 | 11.8 | 18.1 | 11.8 | 2.2 | 3.3 | 2.8 | 2.9 |
| 40-44 | 13.7 | 14.9 | 17.5 | 14.8 | 2.8 | 4.6 | 3.9 | 4.0 |
| 45-49 | 11.8 | 15.2 | 13.0 | 15.6 | 3.6 | 6.7 | 4.2 | 5.2 |
| 50-54 | 10.7 | 16.9 | 12.2 | 16.9 | 3.9 | 7.2 | 4.5 | 7.8 |
| 55-59 | 9.8 | 16.1 | 11.0 | 16.9 | 4.1 | 7.5 | 5.2 | 8.1 |
| 60-64 | 10.7 | 19.6 | 9.8 | 15.5 | 4.6 | 9.2 | 4.7 | 6.8 |
| 65-69 | 10.4 | 17.3 | 10.9 | 20.2 | 4.7 | 10.8 | 4.7 | 6.8 |
| 70-74 | 9.9 | 14.6 | 7.6 | 22.0 | 4.6 | 10.9 | 4.9 | 13.0 |
| 75-79 | 8.2 | 17.7 | 8.5 | 25.2 | 4.7 | 12.5 | 6.7 | 17.2 |
| 80-84 | 7.1 | 20.2 | 8.8 | 27.3 | 5.1 | 15.2 | 5.5 | 15.9 |
| 85+ | 6.6 | 22.2 | 7.4 | 38.0 | 5.4 | 16.3 | 7.4 | 18.2 |

^1^ Rates are per 100,000 and age-adjusted to the 2000 US Standard Population (19 age groups – Census P25-1130) standard. Confidence intervals (Tiwari mod) are 95% for rates.

^2^ Underlying mortality data provided by National Center for Health Statistics (NCHS) - CDC

³ US14: 14 registries in United States; South: 4 registries in Southern States (Georgia and Louisiana); NHW: non-Hispanic white; NHB: non-Hispanic
